# Dropped head sign induced by transdermal application of the dopamine agonist rotigotine in parkinsonian syndrome: a case report

**DOI:** 10.1186/1752-1947-7-174

**Published:** 2013-07-05

**Authors:** Christoph P Dohm, Sonja Gröschel, Jan Liman, Mathias Bähr, Pawel Kermer

**Affiliations:** 1 Department of Neuroradiology, University Medical Center Göttingen, Robert-Koch-Str. 40, Göttingen, 37075, Germany; 2 Department of Neurology, University Medical Center Göttingen, Robert-Koch-Str. 40, Göttingen, 37075, Germany; 3 Department of Neurology, Nordwest-Krankenhaus Sanderbusch, Hauptstr, Sande, 26452, Germany

## Abstract

**Introduction:**

‘Dropped head sign’ relates to a severe disproportionate antecollis in parkinsonism. We present the first report of a rotigotine-induced dropped head sign in a patient with suspected idiopathic Parkinson’s disease, which was later defined as multiple system atrophy. The ‘dropped head sign’ is considered a rare symptom of unknown etiology in parkinsonian disorders, though a disproportionate antecollis is frequently observed in multiple system atrophy. It has also been described as a side effect of dopamine agonist medication with cabergoline and pramipexole. Rotigotine is a transdermally applied, non-ergot dopamine agonist, resulting in a continuous stimulation of dopamine receptors, which is widely used in the treatment of patients with Parkinson’s disease.

**Case presentation:**

We report a case of a 64-year-old Caucasian woman with a rapidly progressive two-and-a-half-year history of a hypokinetic Parkinson’s syndrome with asymmetric development of symptoms and an initially good response to levodopa medication. Due to side effects of other dopamimetic medications the patient was switched to rotigotine medication five weeks before clinical admission. Progressive antecollis without muscle weakness and prominent paraspinal muscle contraction developed within two weeks of treatment and resolved within a week after discontinuation of rotigotine and initiation of levodopa/cabergoline medication.

**Conclusion:**

While the pathophysiology still remains unresolved, this case supports the concept of a dopaminergic imbalance as a cause of certain axial dystonias like disproportionate antecollis including the ‘dropped head sign’. We believe this case is specifically useful for neurologists and general practitioners, as the easily recognizable symptom should prompt a thorough reevaluation of diagnosis and medication in patients with Parkinson’s disease.

## Introduction

The dropped head sign, also referred to as disproportionate antecollis, describes a pronounced anteflexion of head and neck in relation to the trunk without relevant weakness of neck muscles, also named dropped head syndrome by some authors, which usually refers to an isolated weakness of neck extensors [1]. While isolated antecollis is considered a rare etiology, it has been reported in a variety of metabolic and neurological conditions [2,3] as well as neurodegenerative diseases like multiple system atrophy (MSA) and Parkinson’s disease (PD) [4,5]. The etiology of disproportionate antecollis in PD is not understood, but it is considered a form of imbalanced rigidity of neck flexor and extensor muscles or a relative weakness of neck extensor muscles possibly related to neck extensor myopathy. Moreover, disproportionate antecollis was described as a side effect of pramipexole and cabergoline medication in idiopathic Parkinson’s disease, resolving after discontinuation of dopamine agonist treatment [4,6].

Transdermal application of the dopamine agonist rotigotine as a once-daily adhesive patch was introduced into the treatment of PD in Europe in 2006 and became one of the most prescribed new drugs. In 2008, the prescription rate reached 2.8 million defined daily doses in Germany, making it the third most prescribed dopamine agonist following pramipexole and ropinirole [7]. During the last few years, rotigotine was established as a standard component in Parkinson’s medication in the early and late stages of disease. The easy way of application not only offers benefits in terms of medication convenience but also avoidance of side effects related to oral medications, like gastrointestinal side effects. Due to steady-state drug plasma levels it aims at continuous dopaminergic stimulation. As pulsatile dopaminergic stimulation due to multiple daily dosing, as seen with levodopa and most oral medications for PD, is related to motor complications, like fluctuations and dyskinesias, hopes were raised, that transdermal application would also reduce other motor side effects in the treatment of PD. Rotigotine is usually well tolerated; side effects include application site reactions and other adverse effects known from dopamine agonist treatment, that is sleepiness, nausea and arrhythmias.

## Case presentation

A 64-year-old woman was referred to our department after she had subacutely developed a progressive disproportionate antecollis five weeks before admission. Parkinson’s disease had been diagnosed one year before; retrospectively, she had already noticed slowness of movements for the previous one and a half years. Nigrostriatal degeneration was documented by DaTScan™ and, because of asymmetric development of symptoms and good response to levodopa medication, a hypokinetic idiopathic Parkinson’s disease was diagnosed. Despite response to treatment, a satisfactory medication calibration turned out to be difficult and different medications with ropinirole and entacapone had to be terminated due to side effects like therapy-resistant diarrhea and debilitating fatigue. With piribedil medication the patient had developed prominent leg edema, so rotigotine up to 8mg per day was added as a substitute to levodopa, rasagiline and piribedil medication. Within a few days the patient complained about neck pain, stiffness and slight antecollis, which did not improve after discontinuation of piribedil medication. Antecollis symptoms substantially worsened after a further increase of rotigotine dose to up to 16mg per day, progressing into a dropped head sign within two weeks after initiation of rotigotine medication. Clinical deterioration was initially linked to rasagiline, which was subsequently discontinued without effect.

Upon clinical presentation the patient showed a pronounced antecollis with severe neck pain. On clinical examination, an increased activity of head flexor muscles with prominent contraction of levator scapulae muscles was found. With effort the head could temporally be lifted off the chest to some extent, but the patient was able to recline the head when lying down. Besides, the patient showed a slightly reduced facial expression with dysarthrophonia, a right-sided rigor, a slowed gait and a reactive retropulsion of the upper body to be able to view ahead. Differential diagnostic workup of dropped head syndrome, including a magnetic resonance imaging (MRI) scan of her head and neck, a laboratory checkup and examinations for myositis and myasthenic syndromes did not reveal pathological findings. Especially, the head MRI scan showed no signs for atypical Parkinson’s syndrome like putaminal, pontine or cerebellar atrophy, and midbrain ultrasound revealed a hyperechogenic substantia nigra on the left side, supporting the diagnosis of idiopathic PD. Rotigotine dose was reduced and finally discontinued, resulting in marked improvement of antecollis within a week. Because of the aggravation of Parkinson’s symptoms with levodopa monotherapy, a successive co-medication with cabergoline was started following cardiologic clearance without relevant side effects even with doses of up to 6mg per day. Upon external follow-up the patient reported a slowly progressive recurrence of antecollis over a few months unrelated to medication changes, which did not improve after discontinuation of cabergoline medication. MSA was diagnosed due to the additional development of autonomic dysfunction and slight ataxia.

## Discussion

Disproportionate antecollis is altogether considered a rare and inconsistent entity; it has been documented in metabolic and different neurological diseases, including Parkinson’s syndromes, amyotrophic lateral sclerosis, myasthenia gravis and Lambert-Eaton’s syndrome, other forms or myositis and myopathy, facioscapulohumeral dystrophy, chronic inflammatory demyelinating neuropathy, hypokalemic myopathy, hyperparathyroidism and as side effect of neuroleptic or dopamine agonist treatment. Though dropped head sign seems to be more common in connection with Parkinson’s syndromes and was even mentioned in James Parkinson’s famous ‘essay on the shaking palsy’ [8], epidemiological data on prevalence in Parkinson’s patients is scarce. Small Japanese cohort investigations reported a prevalence of up to 6 percent [4,5], which we would consider an optimistic estimate transferred to our European patient cohort. This might also relate to the descriptive definition of a heterogeneous symptom.

While the exact etiology of disproportionate antecollis in parkinsonism is enigmatic, dystonia of neck flexors as well as weakness of neck extensor muscles or an imbalanced rigidity of the latter were described [9]. It is noteworthy that, albeit disproportionate antecollis can indisputably occur in idiopathic Parkinson’s disease, ‘antecollis’ was reported in more than half of the pathologically proven cases of multiple system atrophy and is considered a ‘red flag sign’ for MSA [10]. Interestingly, Quinn and Fujimoto observed an additional torticollis component in MSA patients [4,10] that might support clinical differentiation of idiopathic PD from MSA but it was not found in the presented case. Whether the patient described in this case report developed a torticollis component at recurrence of antecollis cannot be answered, because follow-up visits were conducted in external institutions.

It is suggestive to interpret disproportionate antecollis as a special form of other posture abnormalities that one observes in Parkinson’s patients, like camptocormia (bent spine syndrome) or pleurothotonus (Pisa syndrome), referred to as ‘axial dystonias’ , which might differ in the location of specific lesions that cause a dopaminergic-cholinergic imbalance [11,12]. The dropped head sign has also been reported as a side effect of neuroleptic medication [13] and seems to respond better to levodopa therapy whereas bent spine and Pisa syndrome were not reported as side effects of dopamine agonist treatment, supporting the evaluation of dropped head sign as an independent entity in certain cases.

A small number of reports describe the appearance of dropped head sign caused by ergot and non-ergot dopamine agonist treatment, including cabergoline and pramipexole. While pathophysiology remains unclear, a certain similarity of our case to the case reported by Fujimoto [4] is remarkable; in both cases antecollis due to increased activity of neck flexor muscles developed subacutely over a few weeks following initiation of treatment with cabergoline or rotigotine and symptoms resolved within two weeks after discontinuation of treatment. Considering that dropped head sign was described following neuroleptic and dopamine agonist treatment with structurally different agents of ergot and non-ergot backbone and relapses under levodopa treatment were not observed, a pathophysiological concept involving a dopamine imbalance within the basal ganglia circuit or a differential sensitivity of muscles to dopamine and dopamine agonists, as proposed by Horiuchi and colleagues [14] is suspected. This view is supported by our case, where rotigotine medication might have prematurely triggered a disproportionate antecollis in a predisposed patient with MSA in an early stage of disease. Nevertheless, we cannot rule out a medication interaction-induced side effect of rotigotine, that is with levodopa co-medication.

Furthermore, though different ergot- and non-ergot dopamine agonists can induce dropped head sign, we showed that switching to a different dopamine agonist even at high doses is possible, demonstrating that disposition to develop dropped head sign seems to relate to the specific characteristics of the drug used and not to the whole group. Because the reappearance of disproportionate antecollis a few months later did not improve after discontinuation of cabergoline and marked reduction of levodopa medication (see Additional file 1), a medication side effect seems improbable. Prior high-dose co-medication of levodopa, ropinirole and piribedil medication did not induce an antecollis (see Additional file 1 and Additional file 2), suggesting a rotigotine-specific effect in our patient and not a simple result of cumulative dopaminergic dosage.

Our case further shows, that disproportionate antecollis related to dopamine agonist treatment is not due to fluctuating drug plasma levels or gastrointestinal drug uptake and metabolism.

## Conclusion

In conclusion, awareness to look for the easily recognizable dropped head sign in Parkinson’s patients is advised. One needs to emphasize the highly debilitating nature of this symptom and the risk of incomplete remission due to overstretching of neck extensors and osseous remodeling if recognized too late. Beside the consideration of MSA, the check-up should include recent medication changes, especially neuroleptic agents and dopamine agonists. If dopamine agonists turn out to be the suspected culprits, they should preferably be switched to levodopa therapy. If control of Parkinson’s symptoms is still insufficient, the addition of a different dopamine agonist appears feasible. Transdermal application of rotigotine, though believed to induce less motor complications than other dopamine agonists, can induce a disproportionate antecollis in certain patients.

## Consent

Written informed consent was obtained from the patient for publication of this case report and any accompanying images. A copy of the written consent is available for review by the Editor-in-Chief of this journal.

## Competing interests

CD and PK received travel grants from Abbott Pharma, PK received lecture fees from Boehringer Ingelheim, UCB Pharma and Abbott Pharma.

## Authors’ contributions

CD, SG, MB and PK examined the patient and analyzed and interpreted the patient’s data. JL performed the midbrain ultrasound and interpreted the patient’s MRI scans. CD prepared the manuscript, which was read and approved by all authors.

## Supplementary Material

Additional file 1 Retrospective reconstruction of Parkinson’s disease medication (in mg, DCI – decarboxylase inhibitor) and dropped head sign/antecollis (arbitrary scale; 10 represents maximum symptoms), based on clinical examinations, patient’s descriptions and available medical documentation. Click here for file

Additional file 2 **Extension of Additional file**1**between days 350 and 500.** Retrospective reconstruction of Parkinson’s disease medication (in mg, DCI - decarboxylase inhibitor) and dropped head sign/antecollis (arbitrary scale; 10 represents maximum symptoms). Click here for file

## References

[B1] van de Warrenburg BP Cordivari C Ryan AM Phadke R Holton JL Bhatia KP Hanna MG Quinn NP The phenomenon of disproportionate antecollis in Parkinson’s disease and multiple system atrophy Mov Disord 2007 22 2325 2331 10.1002/mds.21634 17960814

[B2] Kastrup A Gdynia HJ Nagele T Riecker A Dropped-head syndrome due to steroid responsive focal myositis: a case report and review of the literature J Neurol Sci 2008 267 162 165 10.1016/j.jns.2007.09.042 17964603

[B3] Rymanowski JV Twydell PT Treatable dropped head syndrome in hyperparathyroidism Muscle Nerve 2009 39 409 410 10.1002/mus.21092 19208408

[B4] Fujimoto K Dropped head in Parkinson’s disease J Neurol 2006 Suppl 7 VII21 VII26 10.1007/s00415-006-7006-3 17131224

[B5] Kashihara K Ohno M Tomita S Dropped head syndrome in Parkinson’s disease Mov Disord 2006 21 1213 1216 10.1002/mds.20948 16703588

[B6] Taguchi Y Takashima S Tanaka K Pramipexole-induced dropped head syndrome in Parkinson’s disease Intern Med 2008 47 2011 2012 10.2169/internalmedicine.47.1579 19015619

[B7] Schwabe U Paffrath D Arzneiverordnungs-Report 2009 2009 Berlin: Springer

[B8] Parkinson J An essay on the shaking palsy. 1817 J Neuropsychiatry Clin Neurosci 2002 14 223 236 discussion 222 10.1176/appi.neuropsych.14.2.223 11983801

[B9] Yoshiyama Y Takama J Hattori T The dropped head sign in parkinsonism J Neurol Sci 1999 167 22 25 10.1016/S0022-510X(99)00129-X 10500257

[B10] Quinn N Disproportionate antecollis in multiple system atrophy Lancet 1989 1 844 2564927 10.1016/s0140-6736(89)92300-3

[B11] Finsterer J Strobl W Presentation, etiology, diagnosis, and management of camptocormia Eur Neurol 2010 64 1 8 10.1159/000314897 20634620

[B12] Villarejo A Camacho A Garcia-Ramos R Moreno T Penas M Juntas R Ruiz J Cholinergic-dopaminergic imbalance in Pisa syndrome Clin Neuropharmacol 2003 26 119 121 10.1097/00002826-200305000-00004 12782913

[B13] Aries MJ Debruyne H Engelborghs S Le Bastard N Somers N Gorissen D Pickut BA De Deyn PP Reversal of head drop after discontinuation of olanzapine in a DLB patient Mov Disord 2008 23 1760 1762 10.1002/mds.22182 18661562

[B14] Horiuchi M Uehara K Komo T Sugihara H Takahashi Y A case of elderly onset Parkinson’s disease complicated by dropped head syndrome Nippon Ronen Igakkai Zasshi 2001 38 693 695 10.3143/geriatrics.38.693 11605221

