# Supplementary figures and images for: Dropped head sign induced by transdermal application of the dopamine agonist rotigotine in parkinsonian syndrome: a case report
Source: J Med Case Rep. 2013 Jul 5;7:174. doi: 10.1186/1752-1947-7-174 (PMC3708785; doi:10.1186/1752-1947-7-174)

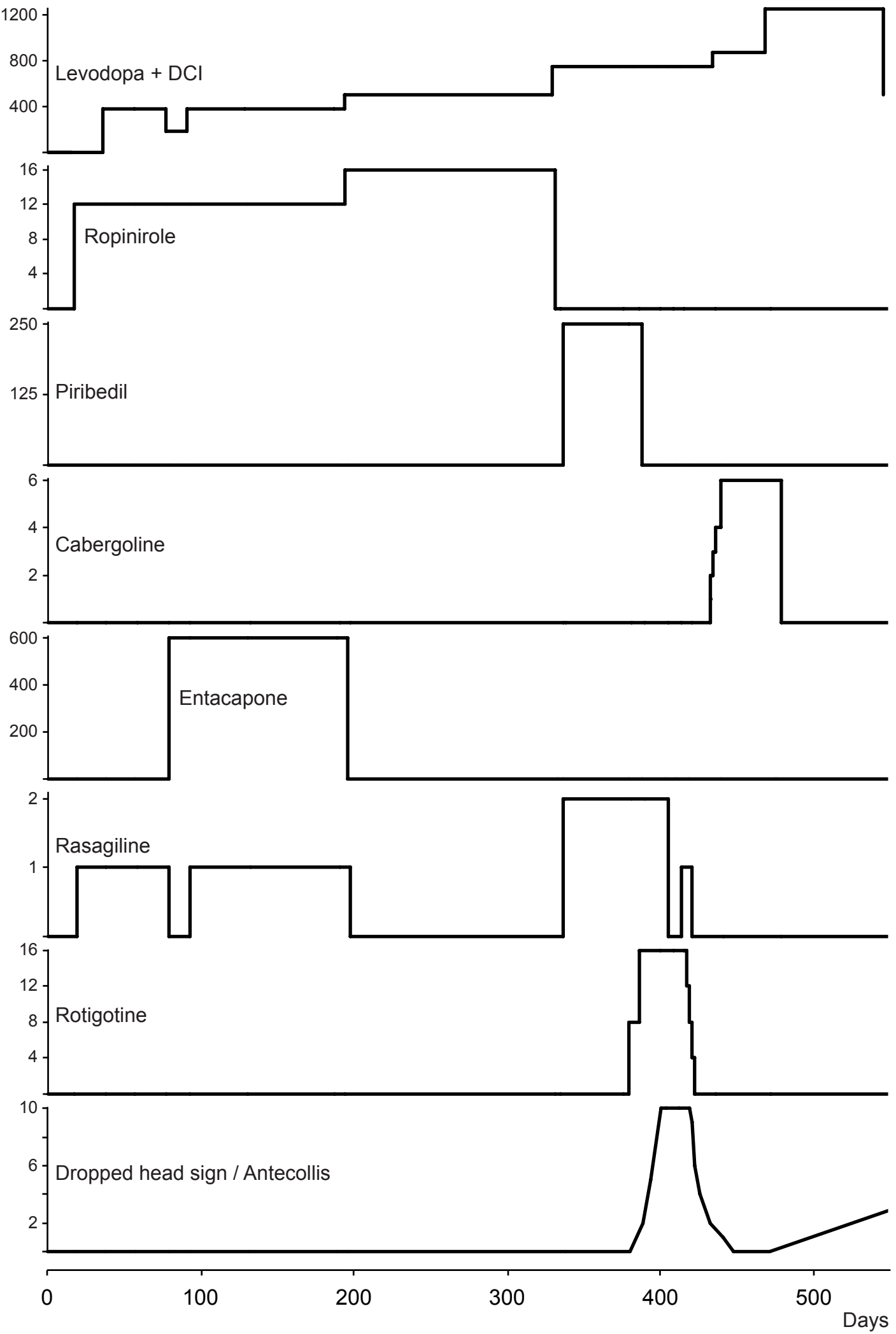

Supplement: Additional file 1 — Retrospective reconstruction of Parkinson’s disease medication (in mg, DCI – decarboxylase inhibitor) and dropped head sign/antecollis (arbitrary scale; 10 represents maximum symptoms), based on clinical examinations, patient’s descriptions and available medical documentation. [file 1752-1947-7-174-S1.pdf]

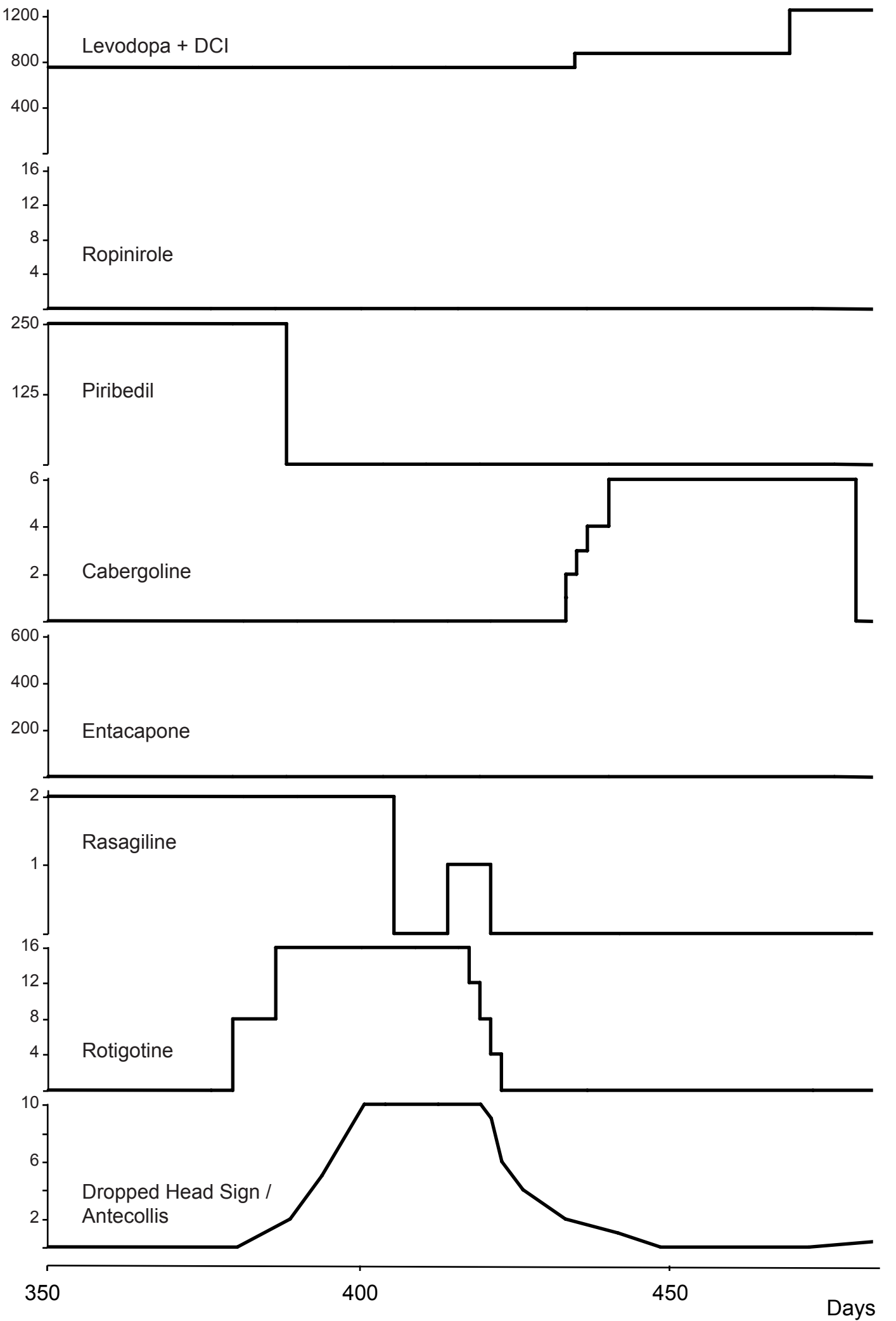

Supplement: Additional file 2 — Extension of Additional file1between days 350 and 500. Retrospective reconstruction of Parkinson’s disease medication (in mg, DCI - decarboxylase inhibitor) and dropped head sign/antecollis (arbitrary scale; 10 represents maximum symptoms). [file 1752-1947-7-174-S2.pdf]
